# Enhanced Photoelectrochemical Water Oxidation Using TiO_2_-Co_3_O_4_ p–n Heterostructures Derived from in Situ-Loaded ZIF-67

**DOI:** 10.3390/ma16155461

**Published:** 2023-08-04

**Authors:** Chau Thi Thanh Thu, Hyo Jeong Jo, Ganesh Koyyada, Dae-Hwan Kim, Jae Hong Kim

**Affiliations:** 1 Department of Chemical Engineering, Yeungnam University, 214-1, Daehak-ro 280, Gyeongsan 712-749, Republic of Korea; chauthuy041091@gmail.com; 2 Division of Energy Technology, Daegu-Gyeongbuk Institute of Science and Technology (DGIST), Daegu 42988, Republic of Korea; 3kubsal@dgist.ac.kr (H.J.J.); monolith@dgist.ac.kr (D.-H.K.)

**Keywords:** Co_3_O_4_-TiO_2_, ZIF-67, p–n junction, photoelectrochemical water splitting, photocurrent

## Abstract

Exposing catalytically active metal sites in metal–organic frameworks (MOFs) while maintaining porosity is beneficial for increasing electron transport to achieve better electrochemical energy conversion performance. Herein, we propose an in situ method for MOF formation and loading onto TiO_2_ nanorods (NR) using a simple solution-processable method followed by annealing to obtain TiO_2_-Co_3_O_4_. The as-prepared TiO_2_-ZIF-67 based photoanodes were annealed at 350, 450, and 550 °C to study the effect of carbonization on photo-electrochemical water oxidation. The successful loading of ZIF-67 on TiO_2_ and the formation of TiO_2_-Co_3_O_4_ heterojunction were confirmed by XRD, XPS, FE-SEM, and HRTEM analyses. TiO_2_-Co_3_O_4_-450 (the sample annealed at 450 °C) showed an enhanced photocurrent of 2.4 mA/cm^2^, which was 2.6 times larger than that of pristine TiO_2_. The improved photocurrent might be ascribed to the prepared p–n heterostructures (Co_3_O_4_ and TiO_2_), which promote electron–hole separation and charge transfer within the system and improve the photoelectrochemical performance. Moreover, the preparation of Co_3_O_4_ from the MOF carbonization process improved the electrical conductivity and significantly increased the number of exposed active sites and enhanced the photoresponse performance. The as-prepared ZIF-67 derived TiO_2_-Co_3_O_4_ based photoanodes demonstrate high PEC water oxidation, and the controlled carbonization method paves the way toward the synthesis of low-cost and efficient electrocatalysts.

## 1. Introduction

Photoelectrochemical (PEC) water splitting is considered one of the most promising strategies for mitigating global fossil fuel shortages and addressing environmental issues [1,2,3]. The choice of semiconductor is critical in enhancing the PEC performance. However, semiconductors employed in PEC water splitting suffer from drawbacks, such as the recombination of photogenerated electron–hole pairs and a comparatively higher band gap, which limit the solar energy conversion efficiency. In this context, titanium dioxide (TiO_2_) has gained considerable attention as a prospective semiconductor for PEC water splitting owing to its perfect band-edge position, environmentally benign features, desired photocorrosion resistance, and cost-effectiveness [1,4]. Despite these advantages, the comparatively larger bandgaps of the rutile (3.0 eV) and anatase (3.2 eV) phases, slow oxygen evolution reaction (OER) kinetics, and a higher charge recombination have limited the use of TiO_2_ in PEC [4]. Several strategies have been proposed to overcome these limitations, including the use of metal oxides [5], heterojunctions [6], surface modification [7], introduction of defects [8], and quantum dots [4].

Among the aforementioned strategies, combining TiO_2_ photoelectrodes with a particular metal oxide is considered an effective method to enhance the PEC performance [9]. A suitable metal oxide with TiO_2_ heterostructure can improve the interfacial electric field and charge carrier migration [10]. Zhang et al. reported that the distinct rod-like structure of TiO_2_ is beneficial for fabricating heterojunctions owing to the large operating surface area of TiO_2_ [11]. Pan et al. found that the photocurrent density of the ZnO/TiO_2_ heterostructure was 3.8 times higher than that of pristine TiO_2_ owing to improved electron–hole separation [12]. Compared to ZnO, Co_3_O_4_ exhibits excellent photocatalytic properties owing to its absorption properties in the visible range [13,14,15]. Several strategies have been used to prepare Co_3_O_4_/TiO_2_ nanostructures. Xian et al. reported a solvothermal technique to prepare 0D/2D Co_3_O_4_/TiO_2_ nanostructures that exhibited enhanced photocatalytic performance [16]. Wang et al. reported a hydrothermal method for preparing Co_3_O_4_ QD/TiO_2_ nanobelts capable of improved water oxidation [17].

Metal–organic frameworks (MOFs) have become promising sacrificial templates for preparing metal oxide-based nanomaterials [18,19] because of the opportunity for property tenability with careful selection of functionalized organic linkers and metal ions [20,21]. Ordered MOF structures at the molecular level with uniform metal ion and organic linker distribution can avoid the regrowth and aggregation (inactivation) of metal/metal oxides during PEC water oxidation [5,22]. In particular, metal oxides derived from hollow MOF structures have porous walls; the large cavities allow an efficient diffusion of the ions and electrolytes, thereby exposing the active sites and minimizing the electron transfer resistance. In addition, the improved conductivity and O_2_ adsorption capacity of MOF-derived metal oxides further increase their electrocatalytic performance [18,23].

Zeolitic imidazolate frameworks (ZIFs) are a large MOF subclass, among which ZIF-67 is one of the most characteristic members [23] with self-assembled Co and imidazole links [24] Du et al. successfully prepared PtCo@NC via the thermal decomposition of Pt@ZIF-67, demonstrating its high catalytic activity and excellent durability in ORR [25]. Sun et al. successfully prepared Ni_2_P/CoN-PCP catalysts from the carbonization of MOF materials, which exhibited remarkable catalytic activity owing to excellent electrical conductivity, exposure of a large number of active sites, improved surface area, and porosity [26]. Recently, Qijia et al. prepared Co_3_O_4_ loaded photoanodes using a GIF-67 templating process by changing the soaking time of TiO_2_ based photoanode in MOF solution. It showed 1.65 higher photocurrent than the pristine TiO_2_ [27]. These previous studies suggest that the fabrication of MOF-derived Co_3_O_4_/TiO_2_ heterostructures is a viable option to enhance PEC water splitting.

**Scheme 1:**
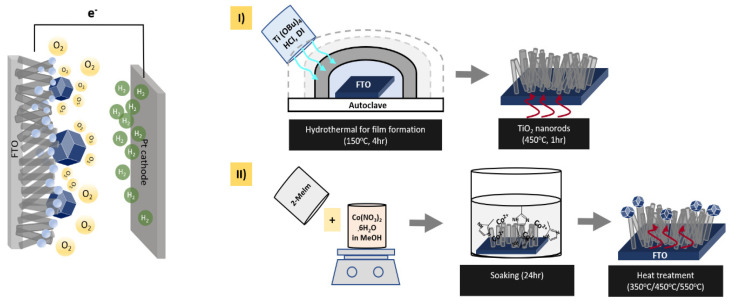
(I) Preparation of the TiO_2_ nanorod film (II) Preparation of the TiO_2_-Co_3_O_4_ nanostructure photoanode for photoelectrochemical water splitting.

Herein, we successfully prepared in situ ZIF-67 loaded TiO_2_ nanorod (NR)-based photoanodes through a simple solvothermal strategy and studied the effect of carbonization on the PEC water oxidation performance by subjecting the photoanodes to carbonization at 350, 450, and 550 °C. The prepared photoanodes were characterized, and their PEC water oxidation performances were systematically compared. Among the four photoelectrodes, the highest photocurrent (2.4 mA·cm^−2^) was observed for TiO_2_-Co_3_O_4_-450 (the sample subjected to carbonization at 450 °C), which was 2.6 times higher than that observed for pristine TiO_2_. The preparation of photoelectrodes is illustrated in Scheme 1.

## 2. Experimental Section

### 2.1. Preparation of Rutile TiO_2_ Film (TiO_2_)

Fluorine-doped tin oxide (FTO)-coated glasses (1.5 mm × 2.5 mm, 8 Ω/cm^2^) were ultrasonically cleaned using detergent, Milli-Q water, ethanol, and acetone, respectively. The TiO_2_ film was synthesized via a previously reported hydrothermal method, with some modifications [28]. In particular, 1.32 mL of TBOT was added dropwise to an 80 mL solution containing equal volumes of HCl (35%) and Milli-Q water under continuous and vigorous magnetic stirring until the solution turned transparent. The solution was then transferred into a 100 mL Teflon-lined stainless steel autoclave. Eight pieces of the FTO glass were held in an upright position using a custom-made Teflon holder and were immersed in the solution and heated to 150 °C in an oven for 4 h. The FTO glass containing the TiO_2_ film was cooled and subsequently rinsed thoroughly with Milli-Q water and ethanol. It was then sintered at an elevated temperature of 450 °C for 1 h in air.

### 2.2. Preparation of TiO_2_-ZIF-67

ZIF-67 was prepared via a previously reported procedure [29]. Briefly, 1.164 g of Co(NO_3_)_2_.6H_2_O was dissolved in 200 mL of methanol and stirred at room temperature to form a “pink A” solution, and the TiO_2_ films were soaked in the pink A solution. Similarly, 0.985 g of Melm was dissolved in 100 mL methanol to form a “transparent B” solution. The transparent B solution was added dropwise to the pink A solution containing the TiO_2_ films, and the mixture was vigorously stirred at room temperature for 24 h. The TiO_2_-ZIF-67 films were thoroughly rinsed with methanol to remove inorganic residues and dried at 50 °C for 1 h in an oven.

### 2.3. Preparation of TiO_2_-Co_3_O_4_

The TiO_2_-ZIF-67 thin films were heated at a ramping speed of 3 °C/min and sintered at 350, 450, and 550 °C in air for 3 h. The three Co_3_O_4_ samples thus obtained are denoted according to the respective sintering temperatures as TiO_2_-Co_3_O_4_-350, TiO_2_-Co_3_O_4_-450, and TiO_2_-Co_3_O_4_-550.

### 2.4. Preparation of Photoanodes for PEC Tests

Copper wires were attached to the as-prepared photoanodes using silver paint for connectivity. The samples were dried in air for 3 h. Finally, the boundaries of the samples were encased using non-conducting epoxy resin, leaving behind an illuminated area of 1 cm^2^. The samples were then dried in a desiccator for at least 3 h.

## 3. Results and Discussion

TiO_2_-Co_3_O_4_ was prepared via a three-step process as illustrated in Scheme 1. Initially, rutile-phase TiO_2_ nanorods (NR) were grown uniformly on an FTO glass plate via a hydrothermal method. Next, ZIF-67 was directly grown in situ on the TiO_2_ NRs via a simple solution-processable wet chemical method. Finally, TiO_2_-Co_3_O_4_ was prepared by calcination. Three composites, namely TiO_2_-Co_3_O_4_-350, TiO_2_-Co_3_O_4_-450, and TiO_2_-Co_3_O_4_-550, were synthesized by varying the calcination temperature. The photo-electrochemical water oxidation capabilities of the as-synthesized photoanodes were investigated.

### 3.1. XRD Characterization of the Prepared Nanostructures

**Figure 1:**
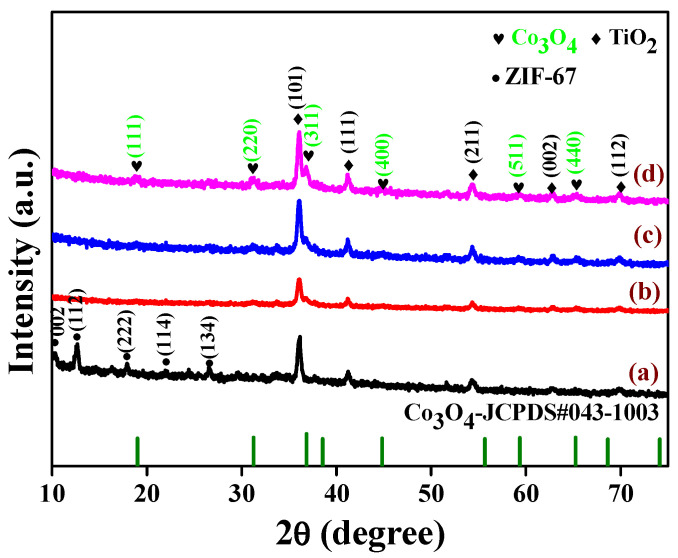
XRD diffraction patterns of (**a**) TiO_2_-ZIF-67, (**b**) TiO_2_-Co_3_O_4_-350 °C, (**c**) TiO_2_-Co_3_O_4_-450 °C, (**d**) TiO_2_-Co_3_O_4_-550 °C.

The crystalline phase and the effect of heating on TiO_2_ and ZIF-67 were investigated using XRD analysis, and the corresponding XRD patterns are depicted in Figure 1 and Figure S1. The diffraction patterns shown in Figure S1 reveal that the TiO_2_ NR was in the rutile phase (JCPDS No. 21-1276), and SnO_2_ was in the tetragonal phase (FTO) (JCPDS. No. 46-1088). The XRD patterns of the TiO_2_-ZiF-67 (Figure 1) photoanode showed new peaks at 10.3°, 12.7°, 17.9°, 22.1°, and 26.6°, corresponding to ZIF-67, thereby confirming that the ZIF-67 was successfully loaded on TiO_2_ [28]. For the electrodes annealed at temperatures 350, 450, and 550 °C, XRD peaks were observed at 19.0°, 31.2°, 36.9°, 41.3°, 54.4°, 62.8°, and 69.9°, corresponding to the planes of (111), (220), (311), (400), (422), (511), and (440), respectively. The observed peaks are all in good agreement with those of Co_3_O_4_ (JCPDS 42-1467) and are well-matched with previous reports [30]. The XRD results indicate that Co_3_O_4_ was formed by the calcination of ZIF-67. Moreover, the characteristic peaks of CoO or other impurities were not observed (Figure 1). The strong intensity peak of the Co_3_O_4_ at 36.9° indicated the (311) plane is the preferred growth orientation.

### 3.2. Morphology Characterization of the Prepared Nanostructures

**Figure 2:**
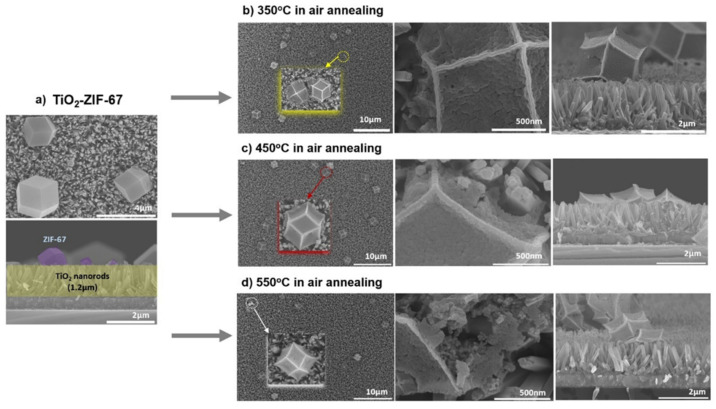
FE-SEM images of the Co_3_O_4_-TiO_2_ nanostructures. (**a**) TiO_2_- ZIF-67, (**b**) TiO_2_-Co_3_O_4_-350, (**c**) TiO_2_-Co_3_O_4_-450, (**d**) TiO_2_-Co_3_O_4_-550.

The surface morphologies and structural patterns of TiO_2_-ZIF-67 and the derived structures were examined via SEM and TEM analyses. Figure 2 shows the NR structure of TiO_2_, which had an average width of ~77.31 nm and a length of ~904.25 nm (Figure S4). Moreover, the top and cross-sectional images shown in Figure 2a confirm that the thickness of the TiO_2_ NR layer was 1.2 μm and that the dodecahedral structure of ZIF-67 was successful loaded [29]. Figure 2b–d show the morphologies of the ZIF-67 after calcination at 350 °C, 450 °C and 550 °C, respectively. As the carbonization temperature increased from 350 to 450 °C, the crystal began to expand at different interfaces, and the interaction with TiO_2_ also was observed to increase (Figure 2b,d). Upon further increasing the carbonization temperature to 550 °C, the MOF framework began to misalign, as shown in Figure 2d. Moreover, the EDS analysis was executed for the TiO_2_-ZIF-67 and TiO_2_-Co_3_O_4_-450 samples, and the results are discussed in Figure S2 and Table S1. Compared to TiO_2_-ZIF-67, the decreased carbon content observed for TiO_2_-Co_3_O_4_-450 might be due to the burning of organic matter lost in the form of CO_2_.

**Figure 3:**
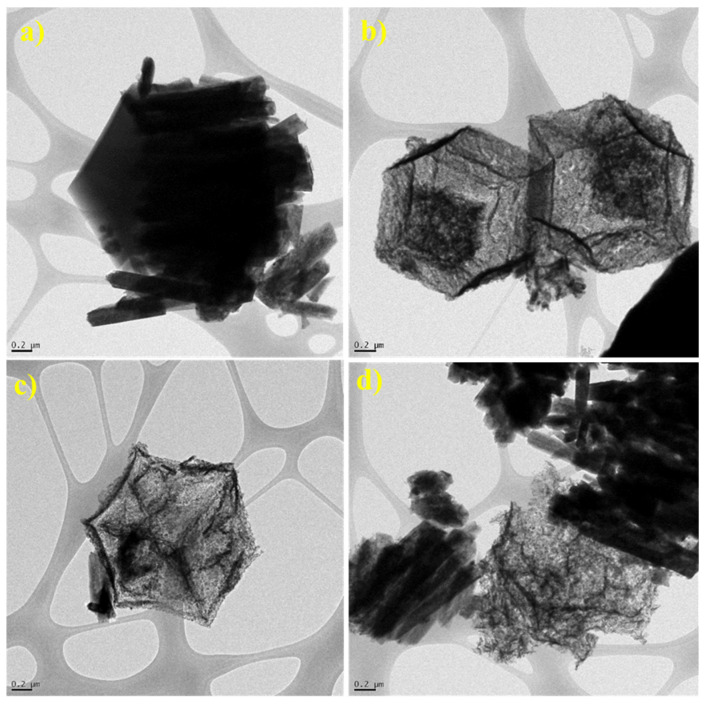
HRTEM images of (**a**) TiO_2_-ZIF-67, (**b**) TiO_2_-Co_3_O_4_-350, (**c**) TiO_2_-Co_3_O_4_-450, (**d**) TiO_2_-Co_3_O_4_-550.

**Figure 4:**
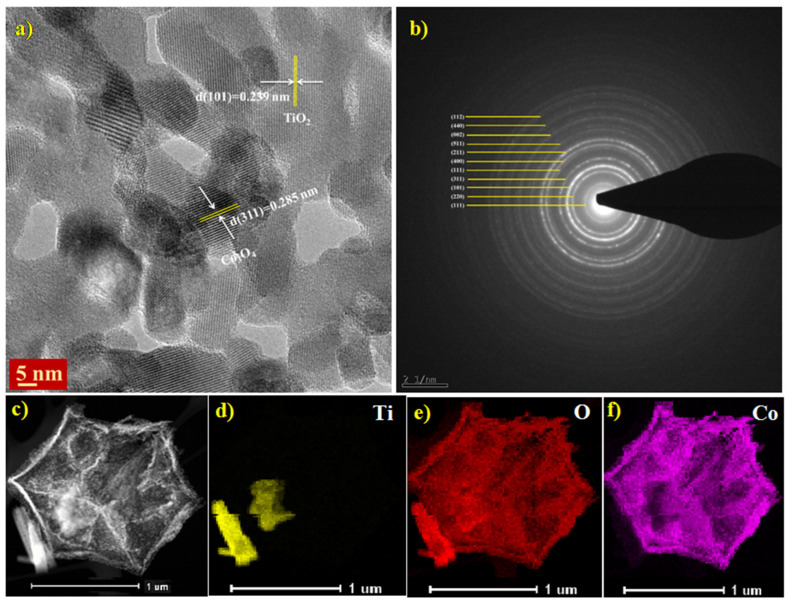
(**a**) HR-TEM. (**b**) The selected area electron diffraction (SAED) pattern. (**c**) HAADF image of the TiO_2_-Co_3_O_4_-450 elemental mapping, (**d**) Ti, (**e**) O, (**f**) Co.

High-resolution transmission electron microscopy (HRTEM) was used to obtain a better understanding of the structural changes due to thermolysis; Figure 3 shows the corresponding HRTEM images. The HRTEM images show the NR and dodecahedral structures of TiO_2_ and ZIF-67 [31], respectively, and are consistent with the FESEM images. As observed in Figure 3b,c, upon calcination of TiO_2_-ZIF-67 at 350 and 450 °C, the hollowed nature inside the dodecahedral increased due to the release of gas like H_2_O and CO_2_ during the calcination process, and the actives sites were exposed. However, further increasing the calcination temperature to 550 °C induced decomposition or misalignment of the nanostructure, as observed in Figure 3d. This decomposition or misalignment can be ascribed to the variance in the thermal expansion coefficients: the contact between dissimilar metals (Ti, Co) increased with an increase in the temperature. The HRTEM image of TiO_2_-Co_3_O_4_-450 (Figure 4a) showed lattice spacing of 0.239 and 0.285 nm attributed to the (311) and (101) planes of TiO_2_ and Co_3_O_4_, respectively [32,33]. Moreover, the selected area electron diffraction (SAED) pattern, displayed in Figure 4b, indicated the multi-crystalline nature of TiO_2_-Co_3_O_4_-450, which correlated with the XRD results. The HAADF spectra of TiO_2_-Co_3_O_4_-450 (Figure 4c–f) illustrate the presence of Co, Ti, and O elements. The obtained results further support the successful formation Co_3_O_4_ metal oxide.

**Figure 5:**
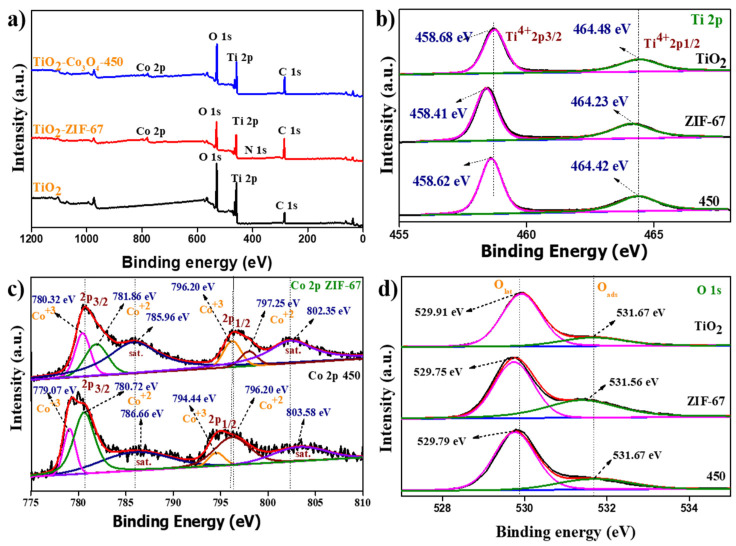
(**a**) XPS survey scan; (**b**) Ti 2p, (**c**) Co 2p, and (**d**) O 1s of TiO_2_-ZIF-67 and TiO_2_-Co_3_O_4_-450.

XPS analysis was conducted to further elucidate the elemental surface composition and electronic state alteration due to thermolysis, and the obtained results are depicted in Figure 5. XPS survey scans of pristine TiO_2_, TiO_2_-ZIF-67, and TiO_2_-Co_3_O_4_-450 are presented in Figure 5a. The presence of Co and Ti in the TiO_2_-ZIF-67 sample indicates the successful decoration of ZIF-67. Furthermore, upon carbonization at 450 °C (TiO_2_-Co_3_O_4_-450), the N element peak disappeared from the TiO_2_-Co_3_O_4_-450 survey scan spectrum, possibly due to ligand loss. These results are consistent with the SEM-EDX analysis results. The XPS spectra of Ti 2p of the as-prepared samples are described in Figure 5b. The binding energies (BE) at 464.44 eV and 458.72 eV were ascribed to the Ti 2p_1/2_ and Ti 2p_3/2_, respectively. The change among these peaks was 5.8 eV, which confirmed the presence of the Ti^+4^ electronic state in TiO_2_ [27,34]. As revealed in Figure 5c, the Co 2p peaks of the TiO_2_-ZIF-67 and TiO_2_-Co_3_O_4_-450 samples showed four peaks attributed to the 2p_1/2_ and 2p_3/2_ doublets and their respective satellite peaks positioned at higher binding energies. The characteristic fitting peaks of TiO_2_-ZIF-67 at 780.32 and 796.20 eV related to Co^+3^, and the 781.86 and 797.25 eV peaks were ascribed to Co^+2^. The Co 2p Co^+3^ and Co^+2^ peaks of the TiO_2_-Co_3_O_4_-450 sample appeared at 779.07/794.44 eV and 780.72/796.20 eV, respectively. The ratio of Co^3+^/Co^2+^ in TiO_2_-Co_3_O_4_-450 was higher compared to the ZIF-67. It can be attributed to the partial ligand damage due to carbonization, which destroys the coordination bond of Co−N and boosts the creation of Co^3+^. The improved Co^3+^ intensity in TiO_2_-Co_3_O_4_-450 indicated better electrochemical activity [35,36]. The O1s XPS spectra of pristine TiO_2_, TiO_2_-ZIF-67, and TiO_2_-Co_3_O_4_ 450 are shown in Figure 5d. All of these samples’ O 1s spectra showed two fitted peaks [37]. The peak appearing at ~529.91 eV was recognized as the lattice oxygen (O_lat_), and the 531.67 eV peak was recognized as the adsorbed oxygen (O_ads_). In general, O_lat_ is more active and significant in redox reactions, whereas O_ads_ depends strongly on the oxidative properties of the catalyst [38]. Additionally, the O_ads_ species concentration is related to the density of oxygen vacancies. Hence, the relative ratios of the O_ads_ peak to the O_lat_ of TiO_2_, TiO_2_-ZIF-67, and TiO_2_-Co_3_O_4_-450 were estimated as 0.26, 0.56 and 0.32, respectively. The higher relative ratio of TiO_2_-Co_3_O_4_-450 further supported the higher catalytic water oxidation property [35].

**Figure 6:**
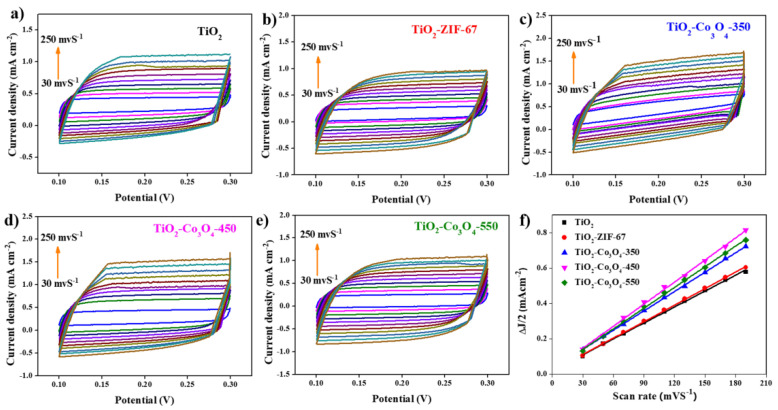
Electrochemical active surface area test (ECSA) of (**a**) TiO_2_, (**b**) TiO_2_-ZIF-67, (**c**) TiO_2_-Co_3_O_4_-350, (**d**) TiO_2_-Co_3_O_4_-450, and (**e**) TiO_2_-Co_3_O_4_-550; (**f**) electrochemical double layer capacity (C_dl_).

To further understand the effect of carbonization temperature on the conductivity of TiO_2_-Co_3_O_4_, an electrochemical double-layer capacitance (C_dl_) experiment was performed, and the electrochemical active surface area (ECSA) was estimated for all of the materials [39,40]. Figure 6a–e display the cyclic voltammetry of TiO_2_, TiO_2_-ZIF-67, TiO_2_-Co_3_O_4_-350, TiO_2_-Co_3_O_4_-450 and TiO_2_-Co_3_O_4_-550, respectively, with changing scan rate from 30 mVS^−1^ to 250 mVS^−1^. This series of experiments was performed to extract the linear relationship of difference in current density (Δj/2) with scan rate. As observed from Figure 6f, TiO_2_, TiO_2_-ZIF-67, TiO_2_-Co_3_O_4_-350, TiO_2_-Co_3_O_4_-450 and TiO_2_-Co_3_O_4_-550 photoelectrodes showed 0.03. 0.031, 0.036, 0.041 and 0.039 mFcm^−2^, respectively. The obtained results suggest that the TiO_2_-Co_3_O_4_-450 photoanode has a larger active electrochemical surface area; it thereby exposes more active sites compared to its counterparts. Hence, the catalytic properties of TiO_2_-Co_3_O_4_-450 could be ascribed to the higher specific surface area.

**Figure 7:**
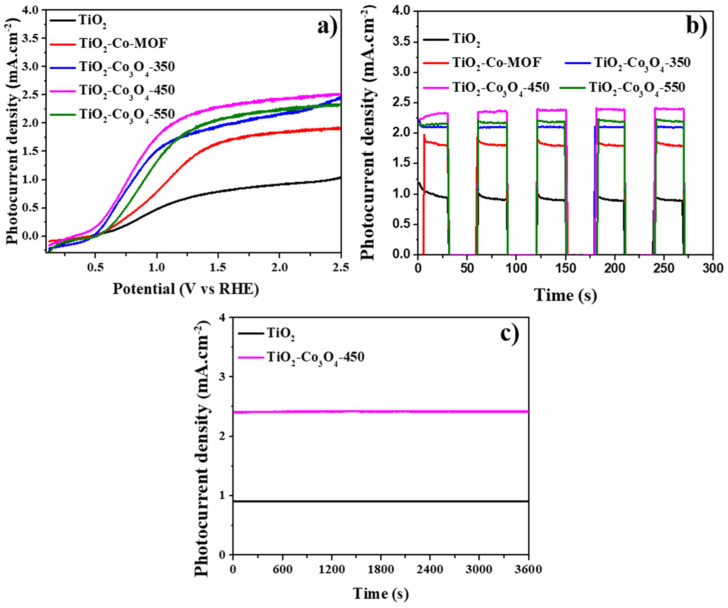
(**a**) LSV plots. (**b**) Transient photocurrent density plots at 1.85 V vs. RHE of all the photoelectrodes. (**c**) Stability test of pristine TiO_2_ and TiO_2_-Co_3_O_4_-450 at 1.85 V vs. RHE.

PEC water oxidation performances of the as-prepared photoelectrodes (TiO_2_, TiO_2_-ZIF-67, TiO_2_-Co_3_O_4_-350, TiO_2_-Co_3_O_4_-450, and TiO_2_-Co_3_O_4_-550) were analyzed using a three-electrode setup under an illumination of 1.5 G at 100 mW/cm^−2^ with 0.5 M Na_2_SO_4_ solution. To study the effect of carbonization temperature on the TiO_2_-ZIF-67 photoanodes, we set the calcination temperatures to 350, 450, and 500 °C, and the prepared photoanodes were named TiO_2_-Co_3_O_4_-350, TiO_2_-Co_3_O_4_-450, and TiO_2_-Co_3_O_4_-550, respectively. Figure 7a demonstrates the linear sweep voltammetry (LSV) results for the as-prepared photoanodes. The TiO_2_-ZIF-67 photoanode showed a photocurrent of 1.8 mA/cm^2^ at 1.85 V vs. RHE, which was twice the photocurrent of the pristine TiO_2_-based photoanode (0.9 mA/cm^2^ at 1.85 V vs. RHE). The increase in photocurrent upon the introduction of ZIF-67 might be due to the improved interaction with molecules of water and its intermediates, which increases the charge transfer between the electrolyte and photoanode interfaces. Upon calcination at 350 and 450 °C, the photocurrent further increased, respectively, to 2.1 and 2.4 mA/cm^2^ at 1.85 V vs. RHE. This increase in the photocurrent can be ascribed to the formation of p-type porous Co_3_O_4_ metal oxide. As observed in the XRD and HRTEM studies, the temperature treatment at 350 and 450 °C resulted in the enhanced particle size along with an increase in the size of the cavity, which facilitated the efficient diffusion of ions and electrolytes. Therefore, the active sites are exposed, thereby promoting interactions with water and its intermediates. Consequently, the electron–hole separation increases at the catalyst interface, leading to increased water oxidation. The photocurrent decreased to 2.2 mA/cm^−2^ upon further heating to 550 °C, which can be attributed to the damaged morphology, as observed in the TEM analysis [6,41]. Moreover, the observed onsite potentials of the as-prepared photoelectrodes of TiO_2_, TiO_2_-ZIF-67, TiO_2_-Co_3_O_4_-350, TiO_2_-Co_3_O_4_-450, and TiO_2_-Co_3_O_4_-550 were 0.59, 0.56, 0.50, 0.46 and 0.55 V vs. RHE, respectively. The enhancement in the photocurrent and the decline in the onsite potential of TiO_2_-Co_3_O_4_ compared to those of TiO_2_-ZIF-67 further demonstrate the advantage of the controlled carbonization process of ZIF-67 in achieving higher PEC water oxidation [42].

Chronoamperometry (CA) analysis was performed with chopped illumination at 1.85 V vs. RHE to better understand the effect of the MOF and its derivatives on the stability and photoresponse of TiO_2_ with respect to time. As shown in Figure 7b, the photocurrent was immediately improved after irradiation and suddenly fell to zero after the irradiation was stopped, suggesting a reproducible current for the prepared photoanodes. These results demonstrated the reproducible nature of the as-prepared photoanodes. The photoresponse of TiO_2_-Co_3_O_4_-based photoanodes was higher than those of TiO_2_-ZIF-67 and pristine TiO_2_, suggesting reduced recombination after carbonization. The observed photocurrent trend in the CA analysis is consistent with the LSV results. Moreover, we performed a one-hour continuous illuminated CA analysis of the pristine TiO_2_ and TiO_2_-Co_3_O_4_-450 photoanodes to assess the durability of the prepared electrodes. As shown in Figure 7c, TiO_2_-Co_3_O_4_-450 photoanodes exhibited 99% of their initial performance even after 1 h of continuous illumination, which is comparable to the stability of TiO_2_. Further, using SEM, we analyzed the morphological changes in TiO_2_-Co_3_O_4_-450 photoanode after 1 h of a stability experiment, and the corresponding SEM images are included in Figure S3.

**Figure 8:**
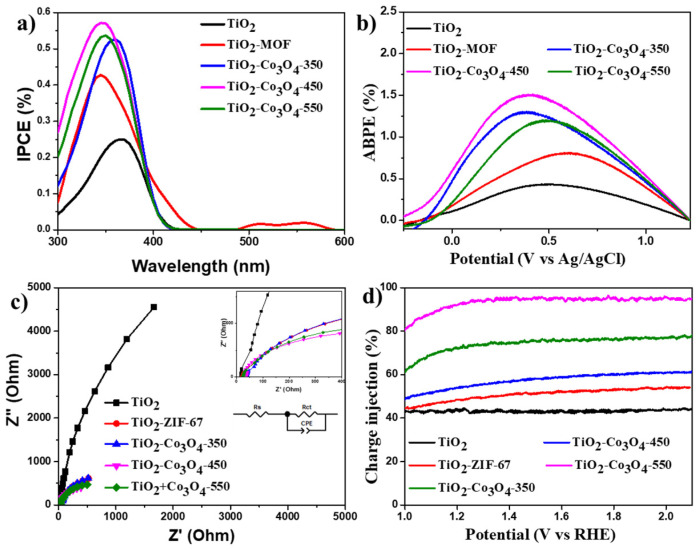
(**a**) Incident photon-to-current conversion efficiency (IPCE), (**b**) ABPE, (**c**) EIS of TiO_2_, TiO_2_-ZIF-67, TiO_2_-Co_3_O_4_- 350 °C, TiO_2_-Co_3_O_4_-450 °C and TiO_2_-Co_3_O_4_-550 °C (upper-right insert figure is the enlargement of high-frequency impedance, and the other insert figure is the equivalent circuit model). (**d**) Charge injection efficiencies of the samples.

IPCE analyses were performed in the wavelength range of 350–550 nm following eq S1 to better understand the energy conversion efficiency of the prepared photoanode. As shown in Figure 8a, all photoanodes showed the highest IPCE at ~350 nm. The observed highest IPCE values of TiO_2_, TiO_2_-ZIF-67, and its derivatives at 350, 450, and 550 °C were 25, 42, 52, 53, and 57%, respectively. The maximum quantum yield was observed for TiO_2_-Co_3_O_4_-450 and was 2.25 times greater than that of the pristine TiO_2_-based photoanode. The results demonstrate the advantage of TiO_2_ and Co_3_O_4_ heterojunctions in improving the visible light harvesting ability and the separation/transportation of photogenerated charge species.

In addition, we calculated the applied bias potential to the current conversion efficiency (ABPE) from the LSV analysis data using eq S2. As found in Figure 8b, the maximum ABPE of pristine TiO_2_, TiO_2_-ZIF-67, and its derivatives at 350, 450, and 550 °C were 0.42, 0.81, 1.20, 1.48, and 1.30%, respectively. The ABPE of TiO_2_-Co_3_O_4_-450 was 3.5 and 1.8 times higher than pristine that of TiO_2_ and TiO_2_-ZIF-67, respectively, indicating efficient charge separation by the introduction of MOF and calcination [4].

EIS was executed to evaluate the carbonization temperature effects on the characteristics of the interfacial charge transfer resistance and carrier transport capacity. In general, the radius of the semicircle in the EIS fitted plots represents the interfacial charge transfer resistance (Rct), that is, the lower the radius, the lower the Rct. As shown in Figure 8c, the Rct decreasing order was TiO_2_ > TiO_2_-ZIF-67 > TiO_2_-Co_3_O_4_-350 > TiO_2_-Co_3_O_4_-450 > TiO_2_-Co_3_O_4_-550. The lower Rct of the TiO_2_-Co_3_O_4_ based photoanodes compared to the TiO_2_–ZIF-67 and pristine TiO_2_ photoanodes demonstrates the advantage of the carbonization process in improving charge transportation. In particular, TiO_2_-Co_3_O_4_-450 showed the lowest Rct, indicating higher charge separation/migration; this could be a possible reason for the higher photocurrent of the TiO_2_-Co_3_O_4_-450 photoanode compared with other photoanodes. Further, the charge injection efficiency (η_injection_) was estimated for the prepared photoanodes by the hole trapping method using H_2_O_2_ as a sacrificial agent [27]. The formula used for the calculation of η_injection_ is as follows.

(1) $$\eta_{\mathrm{injection}}=J_{\mathrm{photocurrent}}/J_{H_{2}}{{}_{O}}_{{}_{2}}$$

J_photocurrent_ and J_H2O2_ are the photocurrent density with and without H_2_O_2_ in the 0.5 M Na_2_SO_4_ solution. As shown in Figure 8d, η_injection_ of TiO_2_, TiO_2_-ZIF-67, TiO_2_-Co_3_O_4_-350, TiO_2_-Co_3_O_4_-450 and TiO_2_-Co_3_O_4_-550 photoanodes were 44.05, 53.42, 60.21, 94.31 and 77.13% at 1.85 V vs. RHE, respectively. The higher η_injection_ was observed for the TiO_2_-Co_3_O_4_-450 compared to its counterpart photoanodes. The higher η_injection_ means lower recombination and a fast charge transfer process.

**Figure 9:**
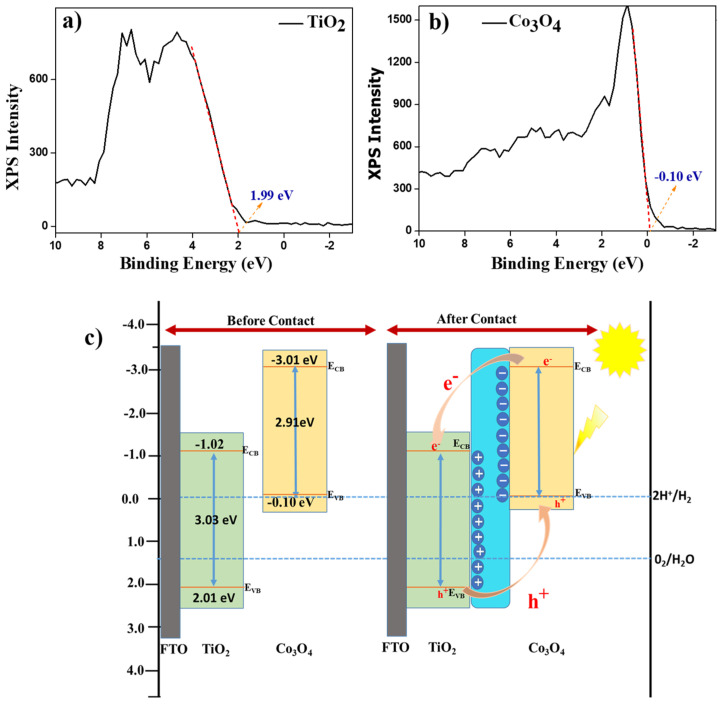
(**a**,**b**) VB-XPS spectra of TiO_2_ and Co_3_O_4_. (**c**) Schematic energy level diagram and charge transfer process for Co_3_O_4_-TiO_2_ heterostructures.

The energy levels of the prepared photoanodes are crucial to understanding the enhanced PEC performance of TiO_2_-CO_3_O_4_ composite material. The valence band (VB) XPS analyses of TiO_2_ and MOF derived Co_3_O_4_ materials were performed, and the obtained plots are illustrated in Figure 9. The valence band maxima (VBM) of individual materials were determined by the extrapolation method. The VBM of TiO_2_ and Co_3_O_4_ were found to be 2.01 and −0.19 eV, respectively, under the Fermi level. The VBM value of Co_3_O_4_ indicated the p-type nature of Co_3_O_4_. Further, the estimated bandgaps from the absorption experiments of TiO_2_ and Co_3_O_4_ were 3.03 and 2.91 eV, respectively. Using the obtained VBM results, the conduction band (CB) edges of TiO_2_ and Co_3_O_4_ were estimated as −1.02 and −3.1 eV, respectively (Figure 9c). Based on the reported p–n junction-based PEC water oxidation mechanism, a space charge layer (SCL) is formed at the interface of p- and n-type semiconductor materials [43,44,45,46]. The generation of the SCL drives the migration of the maximum of charge carriers in opposite directions in p- and n-type materials, which causes the formation of the electric potential at the contacts of the conductors and the p–n junction. Figure 9c illustrates the expected charge transfer methods in the TiO_2_/Co_3_O_4_ based photoanode. The Co_3_O_4_ CB is higher than that of TiO_2_, whereas the Fermi level is less negative (lower). When a heterojunction is formed between TiO_2_ and Co_3_O_4,_ the Fermi level will rearrange and reach the equilibrium level by the diffusion of electron–hole pairs from each material, which leads to the creation of SPL, as mentioned earlier (Figure 9c). Upon irradiation, the generated holes in the VB of TiO_2_ are transferred to Co_3_O_4_ and then moved to water oxidation, while the electrons (photogenerated) from the CB of Co_3_O_4_ are transported to the TiO_2_ and then transferred to the Pt electrode through FTO and back contacts, where H^+^ is reduced and generates H_2_. The electric field formed at the p–n junction of TiO_2_ and Co_3_O_4_ materials could enhance the effective electron–hole pair separation and dramatically lower the rate of recombination [43,47]. This outcome was crucial in improving the photocurrent density observed for TiO_2_/Co_3_O_4_ photoanodes in comparison to TiO_2_ photoanodes. Moreover, the acquired photoelectrochemical performances were compared with the previously reported most similar composites and are listed in Table S2.

## 4. Conclusions

In conclusion, we successfully prepared in situ-loaded TiO_2_-ZIF-67 and TiO_2_-Co_3_O_4_ photoanodes by varying the carbonization temperature from 350 to 550 °C. The effect of carbonization temperature on prepared photoanodes was systematically studied to explore the PEC water oxidation process. Different characterization methods, such as SEM, TEM, XRD, and XPS, disclosed the successful formation of hollow Co_3_O_4_ metal oxides upon thermal treatment. The TiO_2_-Co_3_O_4_-based photoanodes showed higher photocurrent densities and lower onsite potentials than the TiO_2_-ZIF-67 and pristine TiO_2_ photoanodes. In particular, an improved photocurrent of 2.4 mA·cm^−2^ was observed for the TiO_2_-Co_3_O_4_-450 photoanode, which was 2.6 times larger than that of the pristine TiO_2_ photoanode and 1.33 times larger than that of the TiO_2_-ZIF-67 photoanode. The XRD and HR-TEM analyses revealed that the derived Co_3_O_4_ has a larger crystalline size and cavity size than those of ZIF-67, and the crystalline size and cavity size were increased by raising the carbonization temperature from 350 to 450 °C. The increased porous surface and cavity inside the Co_3_O_4_ particles allow for efficient diffusion of ions and electrolytes. Therefore, the number of exposed active sites increases, leading to increased electron–hole separation and transportation. Moreover, impedance analyses revealed that TiO_2_-Co_3_O_4_ has a lower charge transfer resistance than TiO_2_-ZIF-67, which further supports the increased electron separation and transport due to the formation of porous Co_3_O_4_ metal oxide. The increased electron–hole separation, decreased charge transfer resistance, and improved interaction with water molecules and their intermediates are the possible reasons for the increase in the photocurrent of the TiO_2_-Co_3_O_4_ photoanodes. The obtained results indicate the advantage of controlled carbonization of ZIF-67 in improving its catalytic properties and pave the way for the synthesis of better PEC catalysts.

## Data Availability

Data will be made available on request.

## Supplementary Materials

The following supporting information can be downloaded at: https://www.mdpi.com/article/10.3390/ma16155461/s1, General Procedures, Materials, Characterizations, PEC analysis procedure, XRD of pristine TiO_2_ and FTO films, SEM-EDS analysis [27,48,49,50,51,52,53].

## Abbreviations

MOFs, metal-organic frameworks; NR, nanorods; PEC, Photoelectrochemical; ZIFs, Zeolitic imidazolate frameworks; TBOT, titanium butoxide; 2-Melm, 2-Methylimidazole; FTO, Fluorine-doped tin oxide; FESEM, field-emission scanning electron microscopy; FETEM, field-emission transmission electron microscopy; EDS, energy dispersive spectrometry; XRD, X-ray diffraction; XPS, X-ray photoelectron spectroscopy; LSV, Linear sweep voltammetry; EIS, Electrochemical impedance spectroscopy; IPCE, incident photon-to-current conversion efficiency; HRTEM, High-resolution transmission electron microscopy; SAED, selected area electron diffraction; CA, chronoamperometry; ABPE, applied bias potential to the current conversion efficiency.
