# Supplementary material for: Enhanced Photoelectrochemical Water Oxidation Using TiO2-Co3O4 p–n Heterostructures Derived from in Situ-Loaded ZIF-67
Source: Materials (Basel). 2023 Aug 4;16(15):5461. doi: 10.3390/ma16155461 (PMC10420101; doi:10.3390/ma16155461)
Supplement: Supplementary file 1 [file materials-16-05461-s001.zip › materials-2474898-supplementary.pdf]

Supporting Information

Enhanced photo-electrochemical water oxidation using TiO_2-Co_3O_4 p-n heterostructures derived from in situ-loaded ZIF-67

S1. General procedures

S1.1 Materials:

All the chemicals were used directly without further purification. Hydrochloric acid (HCl, 35%) was purchased from OCI Company Ltd.; titanium butoxide (TBOT, 98%) was purchased from Sigma-Aldrich; 2-methylimidazole (2-Melm, 99%) was purchased from Acros Organics; cobalt (II) nitrate hexahydrate ($Co(NO_3)_2.6H_2O$, 97%) was purchased from Shinyo Pure Chemical Company Ltd.; and sodium sulfate anhydrous (Na_2SO_4, 99%) was purchased from Duksan. Milli-Q water (MΩ 18) was used in all the experiments.

S1.2. Characterization:

The morphology and composition of the TiO_2, TiO_2-ZIF-67, and TiO_2 nanostructures with Co_3O_4 were respectively studied using a field-emission scanning electron microscope (FESEM, S4800 Hitachi, Japan) and a field-emission transmission electron microscope (FETEM, Tecnai G2 F20, USA) attached to an energy dispersive spectrometer (EDS). The crystalline structures of the samples were acquired via X-ray diffraction (XRD; X'pert PRO MPD diffractometer, Netherlands) with a PANalytical X' Celerator detector and a Cu Kα (λ = 0.15406 nm) source. The absorption spectra of the newly developed materials were recorded on a Cary 5000 UV-Vis-NIR spectrophotometer (Agilent, USA). The surface elemental compositions and chemical states

were examined via X-ray photoelectron spectroscopy (XPS) using a K-Alpha spectrometer (Thermo Scientific, USA) equipped with a monochromatic Al-Kα X-ray source.

S1.3. PEC tests:

PEC tests were performed using an electrochemical workstation (Zive SP1, WonATech Co., Ltd., Korea) in an electrochemical cell with a three-electrode system: the as-prepared photoanode was used as the working electrode, a platinum mesh was used as the counter electrode, and an Ag/AgCl electrode (in 3 M KCl) placed in a Luggin capillary was used as the reference electrode. The PEC test and other electrochemical measurements were performed using electrodes placed at appropriate positions in a quartz electrochemical cell measuring 50 mm × 50 mm × 20 mm (Hellma, HE.700.000. OG20.) with 0.3 M Na_2SO_4 (pH = 7) as the electrolyte. Linear sweep voltammetry (LSV) curves were recorded in a potential window ranging from −0.5 V to 1.3 V vs Ag/AgCl at a scan rate of 20 mV s^{-1}. Electrochemical impedance spectroscopy (EIS) was performed in the frequency range from 10^{-2} Hz to 10^5 Hz with an amplitude of 5 mV at a bias potential of 0 V vs. Ag/AgCl under solar simulator (AM 1.5G, 100 mW/cm^2) irradiation. The incident photon-to-current conversion efficiency (IPCE) was measured at 1.23 V vs Ag/AgCl using a xenon arc lamp (75 W, Ushio UXL-75XE) filtered using a dual-grafting monochromator and individual filters onto the test devices.

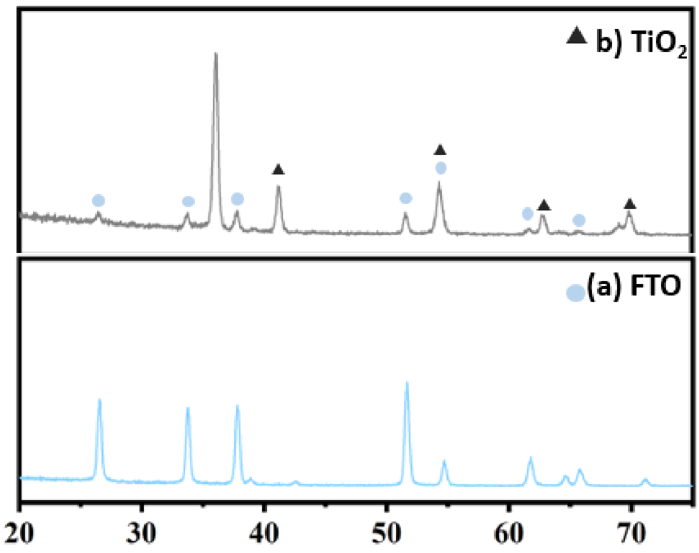

Figure S1. XRD diffraction patterns of (a) FTO(b)TiO_2

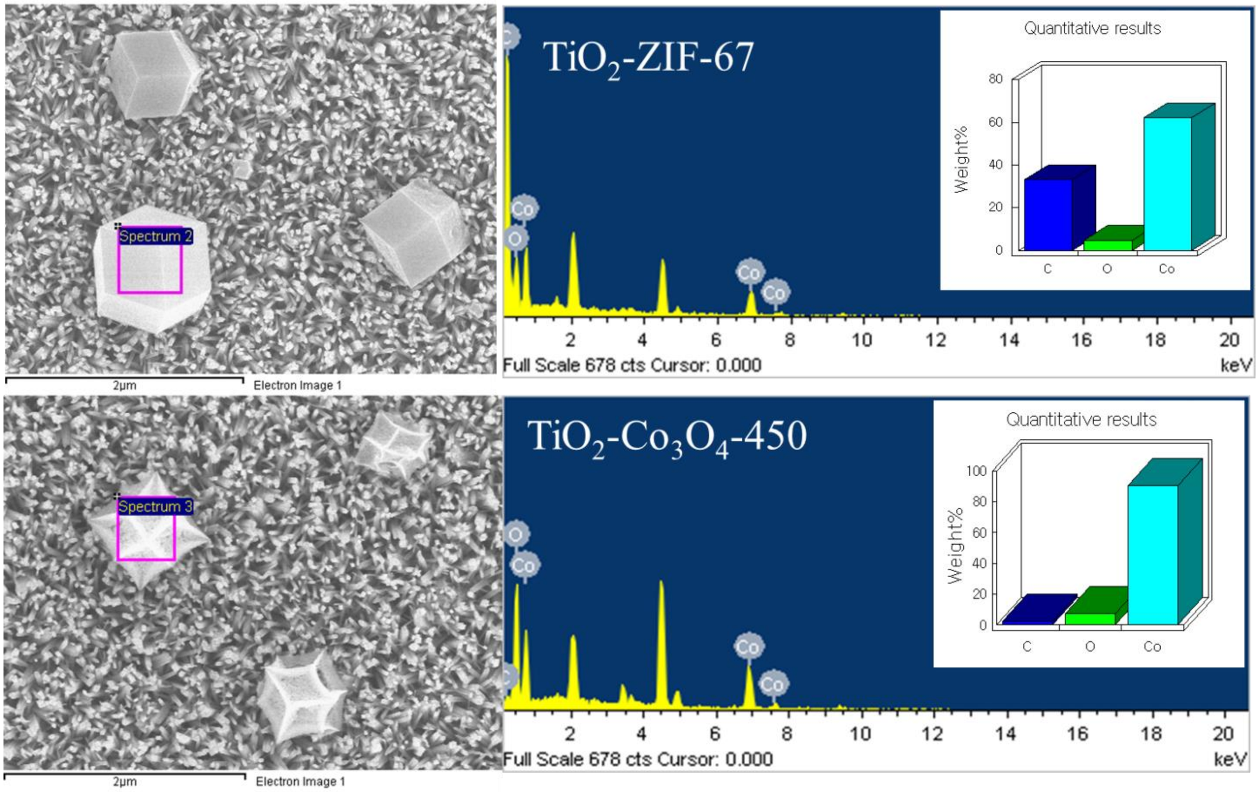

Figure S2. SEM-EDS analysis of ZIF-67 and TiO_2-Co_3O_4-450.

Table S1.EDS-elemental ratio of ZIF-67 and TiO_2-Co_3O_4-450

Elements	ZIF-67		Co_3O_4-450	
	Weight%	Atomic%	Weight%	Atomic%
C	32.98	66.94	2.39	9.09
O	4.80	7.32	7.31	20.89
Co	62.21	25.74	90.30	70.02

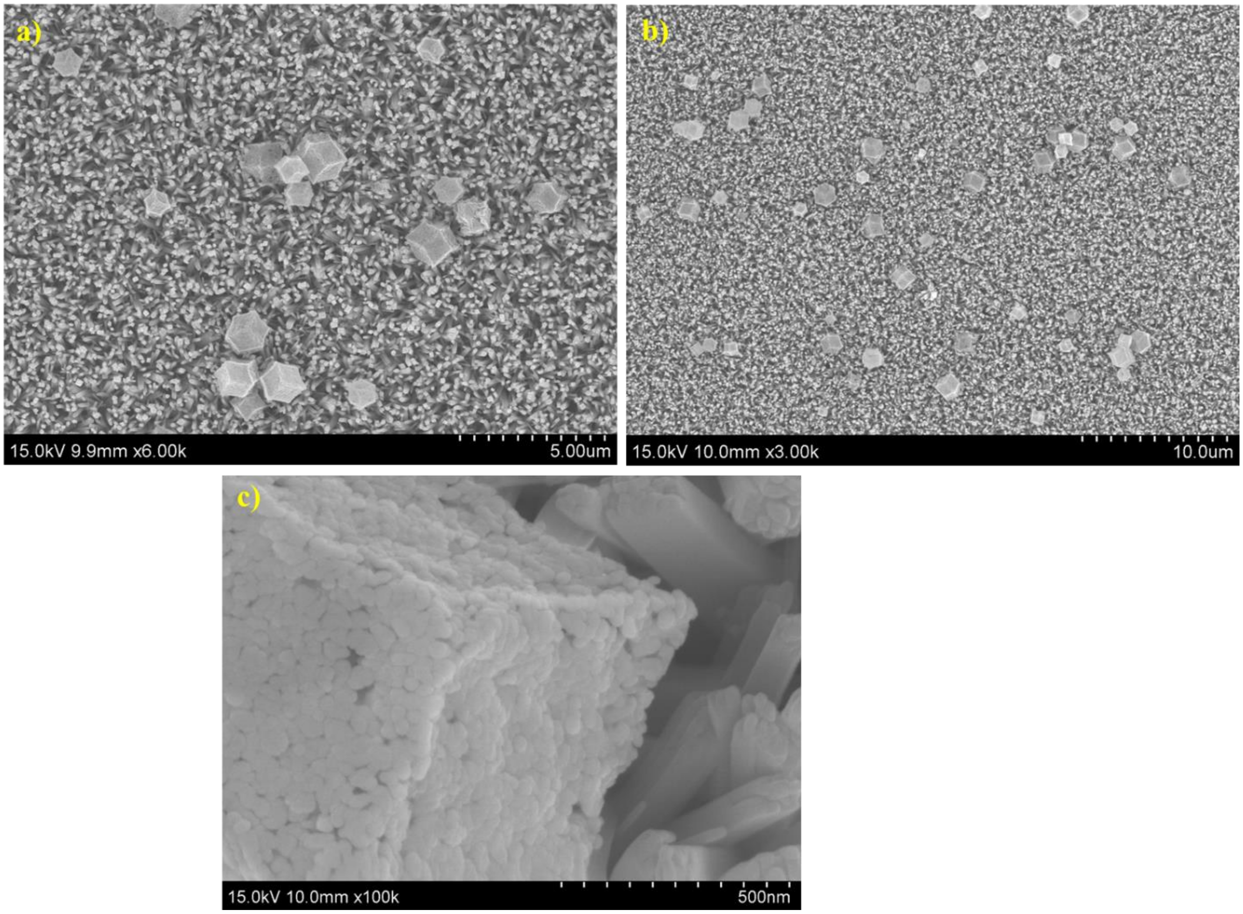

Figure. S3. SEM images of TiO_2-Co_3O_4-450 sample after 1h stability test

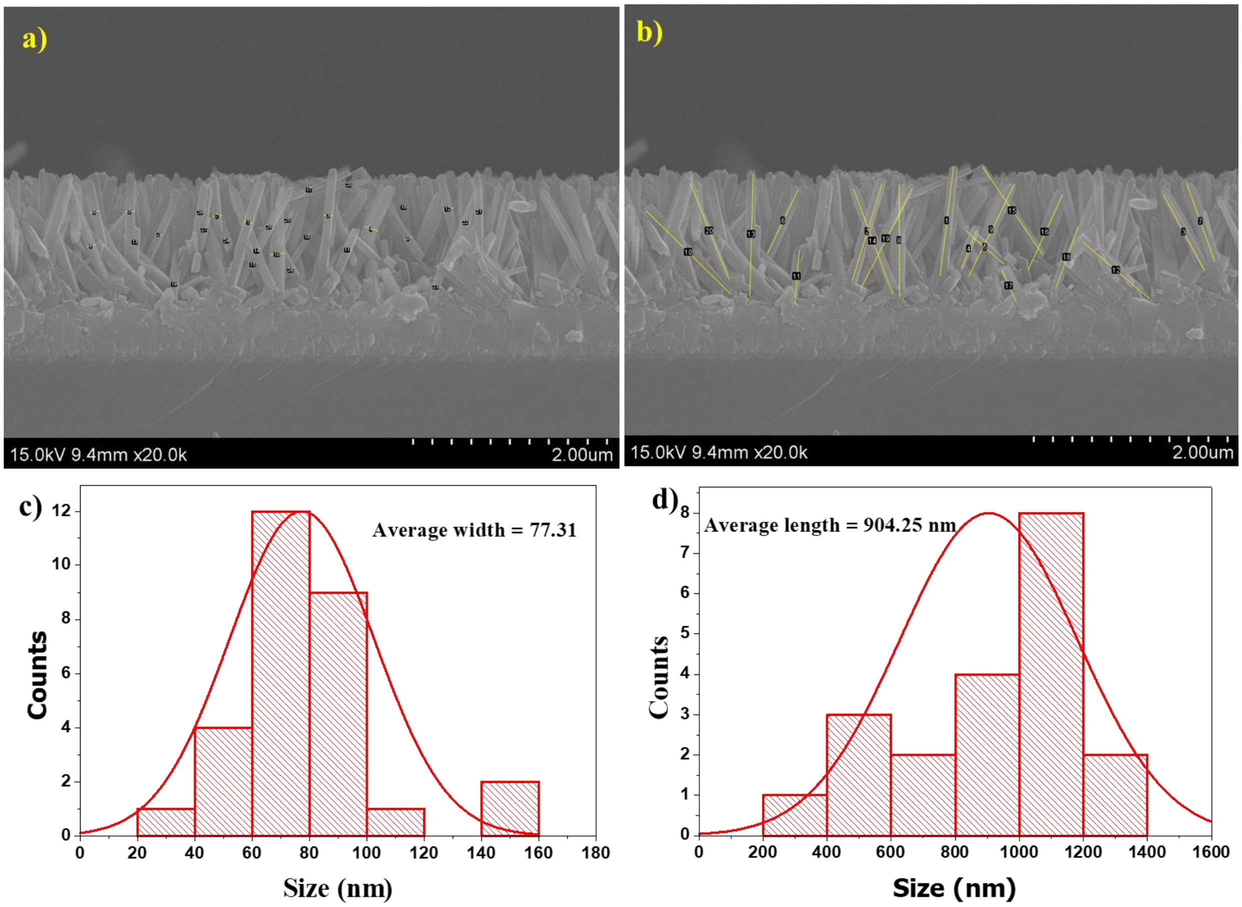

Figure. S4. The Sem and size distribution histogram of TiO_2 nanorod a,c) width of the TiO_2 b,d) Length ot the TiO_2 nanorod.

Table S2. Comparative photoelectrochemical performance of selected TiO_2 or Co_3O_4 nanocomposites

Photoanode	Electrolyte/illumination	Photocurrent	Applied bias (V vs RHE)	Ref
Ag/NH_2-MIL-	0.5 M Na_2SO_4	1.06 mA cm^{-2}	1.23 V vs	[1]

125/TiO_2	Electrolyte/300 W Xe lamp with 100 $mWcm^{-2}$		RHE	
Co_3O_4/TiO_2	1 M NaOH (pH = 14) 300 W xenon lamp (100 $mWcm^{-2}$	1.04 mA cm^{-2}	1.23 V vs RHE	[2]
Co-MOF@TiFe.	1 M NaOH (pH=13.6)/ 100 $mWcm^{-2}$	1.78 mA cm^{-2}	1.23 V vs RHE	[3]
$Co_3O_4/BiVO_4$	0.1M KPi buffer (pH7) 300 W Xe lamp with 100 $mWcm^{-2}$	2.71 mA cm^{-2}	1.23 V vs RHE	[4]
Co_3O_4/P-C_3N_4/TiO_2	0.5 M Na_2SO_4/ 150 W xenon lamp with 100 $mWcm^{-2}$	1.58 mA cm^{-2}	1.23 V vs RHE	[5]
CoNi-MOFs/$BiVO_4$	0.5 M Na_2SO_4/100 $mWcm^{-2}$	3.2 mA cm^{-2}	1.23 V vs. RHE	[6]
NiFe-MOF/TiO_2	0.5 M Na_2SO_4/100 $mWcm^{-2}$	0.77 mA cm^{-2} at	1.23 V (vs. RHE)	[7]
TiO_2-ZIF-67	0.5 M Na_2SO_4/100 $mWcm^{-2}$	1.8 mA cm^{-2}	1.85 V Vs RHE	This work
TiO_2-Co_3O_4-350	0.5 M Na_2SO_4/100 $mWcm^{-2}$	2.1 mA cm^{-2}	1.85 V Vs RHE	This work
TiO_2-Co_3O_4-450	0.5 M Na_2SO_4/100 $mWcm^{-2}$	2.4 mA cm^{-2}	1.85 V Vs RHE	This work
TiO_2-Co_3O_4-550	0.5 M Na_2SO_4/100 $mWcm^{-2}$	2.2 mA cm^{-2}	1.85 V Vs RHE	This work

References

1. Cui, W.; Shang, J.; Bai, H.; Hu, J.; Xu, D.; Ding, J.; Fan, W.; Shi, W. In-situ implantation of plasmonic Ag into metal-organic frameworks for constructing efficient Ag/NH2-MIL-125/TiO2 photoanode. *Chemical Engineering Journal* **2020**, *388*, 124206.

2. Ding, Q.; Gou, L.; Wei, D.; Xu, D.; Fan, W.; Shi, W. Metal-organic framework derived Co3O4/TiO2 heterostructure nanoarrays for promote photoelectrochemical water splitting. *International Journal of Hydrogen Energy* **2021**, *46*, 24965-24976.
3. Li, L.; Zhang, H.; Liu, C.; Liang, P.; Mitsuzaki, N.; Chen, Z. Effect of Co-Based Metal–Organic Framework Prepared by an In Situ Growth Method on the Photoelectrochemical Performance of Electrodeposited Hematite Photoanode. *Energy Technology* **2019**, *7*, 1801069, doi:https://doi.org/10.1002/ente.201801069.
4. Chang, X.; Wang, T.; Zhang, P.; Zhang, J.; Li, A.; Gong, J. Enhanced Surface Reaction Kinetics and Charge Separation of p–n Heterojunction Co3O4/BiVO4 Photoanodes. *Journal of the American Chemical Society* **2015**, *137*, 8356-8359, doi:10.1021/jacs.5b04186.
5. Yu, Z.; Li, Y.; Qu, J.; Zheng, R.; Cairney, J.M.; Zhang, J.; Zhu, M.; Khan, A.; Li, W. Enhanced photoelectrochemical water-splitting performance with a hierarchical heterostructure: Co3O4 nanodots anchored TiO2@P-C3N4 core-shell nanorod arrays. *Chemical Engineering Journal* **2021**, *404*, 126458, doi:https://doi.org/10.1016/j.cej.2020.126458.
6. Zhou, S.; Chen, K.; Huang, J.; Wang, L.; Zhang, M.; Bai, B.; Liu, H.; Wang, Q. Preparation of heterometallic CoNi-MOFs-modified BiVO4: a steady photoanode for improved performance in photoelectrochemical water splitting. *Applied Catalysis B: Environmental* **2020**, *266*, 118513.
7. Cui, W.; Bai, H.; Shang, J.; Wang, F.; Xu, D.; Ding, J.; Fan, W.; Shi, W. Organic-inorganic hybrid-photoanode built from NiFe-MOF and TiO2 for efficient PEC water splitting. *Electrochimica Acta* **2020**, *349*, 136383, doi:https://doi.org/10.1016/j.electacta.2020.136383.
